# Blue Light Receptor WC-2 Regulates Ganoderic Acid Biosynthesis in *Ganoderma lingzhi*

**DOI:** 10.3390/jof11090646

**Published:** 2025-09-01

**Authors:** Yan Xu, Xiong-Min Huang, Zi-Xu Wang, Ying-Jie Zhao, Dong-Mei Lv, Jun-Wei Xu

**Affiliations:** Faculty of Life Science and Technology, Kunming University of Science and Technology, Kunming 650500, China; xuyan@stu.kust.edu.cn (Y.X.); 18287455094@163.com (X.-M.H.); w1192176787@163.com (Z.-X.W.); 20231118007@stu.kust.edu.cn (Y.-J.Z.)

**Keywords:** *Ganoderma lingzhi*, ganoderic acid, white collar 2, blue light induction, biosynthesis

## Abstract

Ganoderic acid (GA) is a key bioactive component with pharmacological properties that is found in *Ganoderma lingzhi*, a renowned medicinal mushroom. Currently, the regulatory mechanisms underlying GA biosynthesis in *G. lingzhi* remain to be further elucidated. In this study, blue light induction was found to significantly enhance the GA content in *G. lingzhi*. To explore the regulatory mechanism of GA biosynthesis in response to blue light, the blue light receptor WC-2 was identified, and its regulatory role was characterized. The deletion of *wc-2* resulted in a significant reduction in both GA content and the accumulation of intermediates compared to the wild-type control strain, largely due to the strong downregulation of key GA biosynthetic genes. Additionally, decreased asexual spore production and reduced expression of sporulation-specific genes were observed with the deletion of *wc-2*. The overexpression of *wc-2* led to greatly enhanced GA accumulation. Under blue light induction, the maximum contents of GA-Mk, GA-T, GA-S, and GA-Me were 2.27-, 2.51-, 2.49-, and 2.08-fold higher, respectively, compared to the control kept in darkness. These results demonstrate that the blue light receptor WC-2 functions as a positive regulator of GA biosynthesis in *G. lingzhi*, influencing the expression of genes involved in GA biosynthesis and asexual spore production, thereby advancing our understanding of the intricate regulatory network of GA biosynthesis.

## 1. Introduction

*Ganoderma lingzhi* is a highly regarded traditional medicinal mushroom used in Asian medicine for over 2000 years to treat various health conditions and promote longevity [1]. Recently, it has garnered increased attention due to ongoing research revealing its novel nutritional and pharmacological properties. Ganoderic acids (GAs) are important bioactive components found in *G. lingzhi*, consisting of highly oxygenated C_30_ lanostane-type triterpenoids with unique bioactivities. They exhibit a wide range of pharmacological effects, including anti-tumor, anti-metastatic, anti-inflammatory, antioxidant, antiviral, anti-aging, anti-hepatotoxic, hypocholesterolemic, and antiglycemic activities [2,3]. For instance, GA-S and GA-Mk trigger apoptosis in HeLa cells, while GA-T and GA-Me suppress the growth and metastasis of lung cancer cells [4,5].

GAs are biosynthesized via the mevalonate/isoprenoid pathway in *G. lingzhi* [6,7]. Several key enzymes involved in lanosterol formation, the basic skeleton of all GAs, have been characterized in *G. lingzhi*, including 3-hydroxy-3-methylglutaryl coenzyme A reductase (HMGR), squalene synthase (SQS), and lanosterol synthase (LS) [3,8]. Following this, lanosterol undergoes a series of reduction, oxidation, and acylation reactions to yield various individual GAs. However, the genes involved in this biotransformation process are not yet fully understood [9,10]. Recent studies have identified cytochrome P450s (CYPs) as essential catalysts for the conversion of lanosterol into various types of GAs, such as CYP5150L8, CYP512U6, and CYP512W2 [11,12,13].

The regulation of GA biosynthesis is predominantly governed by a complex regulatory network [14]. Research has demonstrated that critical genes involved in GA biosynthesis, such as *hmgr*, *ls*, *se*, *sqs*, and *fps*, play crucial roles in its regulation [10,15,16,17,18]. Environmental factors such as temperature, oxygen levels, pH, nitrogen sources, light, and fermentation conditions also significantly influence the regulation of GA biosynthesis [7,14,19,20]. Signaling molecules involved in signal transduction pathways, such as nitric oxide, cyclic adenosine monophosphate, reactive oxygen species, and Ca^2+^, facilitate the regulation of GA biosynthesis [10,21,22,23]. Additionally, several transcription factors that regulate GA biosynthesis have been identified, including AreA, Crz1, LaeA, PacC, and Swi6 [24,25,26,27,28]. Despite existing advances, the regulation of GA biosynthesis remains governed by a complex and not yet fully understood regulatory system. Further exploration of this system would shed light on the intricate regulatory mechanisms controlling GA production in *G. lingzhi*.

Blue light serves as a crucial light signal in the life cycle of fungi, influencing numerous biological processes, such as growth, morphogenesis, and secondary metabolism [29]. In *G. lingzhi*, blue light has been shown to promote fruiting body development, extracellular enzyme activity, and polysaccharide synthesis [30,31]. The WC-2 protein is a critical component of the blue light photoreceptor complex known as the White Collar Complex (WCC), which is primarily responsible for mediating blue light signaling [32]. The WCC complex can directly bind to and activate a range of light-inducible genes, thereby regulating blue light signaling. WC-2 has been cloned and characterized in several fungal species, revealing its significant role in regulating secondary metabolism. For instance, the deletion of *wc-2* in *Neurospora crassa* resulted in the inhibition of photoinduced carotenoid biosynthesis [33]. Similarly, the disruption of WC-2 in *Fusarium graminearum* delayed the early onset of carotenogenesis, impaired sexual development, and derepressed trichothecene production [34]. In *G. lucidum*, the *wc-2* gene was cloned, and sequence analysis revealed that WC-2 functions as a transcription factor containing a GATA-type zinc finger domain [35]. However, it is unclear whether and how WC-2 regulates GA biosynthesis in *G. lingzhi*.

Asexual sporulation also plays a crucial role in GA biosynthesis, as asexual spores are typically enriched with GAs [36]. Notably, a significant positive correlation has been observed between the contents of GAs and the production of asexual spores [9,37]. These findings suggest that promoting asexual sporulation will contribute to regulating the biosynthesis of GAs in *G. lingzhi*. Furthermore, previous studies have demonstrated that exposure to blue light often stimulates asexual sporulation in most fungi, which requires the activity of the WCC complex [38]. However, the role of WC-2 in the regulation of asexual sporulation in *G. lingzhi* remains to be elucidated.

Herein, the blue light receptor WC-2 was identified and its regulatory role in the GA biosynthetic pathway was characterized. The deletion of *wc-2* led to a significant reduction in GA biosynthesis due to the decreased accumulation of intermediates and downregulation of key GA biosynthetic genes. It also resulted in reduced asexual spore production. In contrast, the overexpression of *wc-2* was shown to dramatically enhance GA content. This study provides valuable insight into the regulatory mechanisms underlying GA biosynthesis in *G. lingzhi*.

## 2. Materials and Methods

### 2.1. Strains, Culture Conditions, and Light Treatment

The strains *G. lingzhi* Cas9 and *G. lingzhi* CGMCC 5.616-1 [25] served as the parental strains for constructing Δ*wc-2* and OE*wc-2* strains, respectively. Plasmid construction was carried out using *Escherichia coli* DH5α.

For the solid cultivation of *G. lingzhi*, equal amounts of pre-grown mycelia were inoculated onto the center of cellophane-lined PDA agar plates and incubated at 30 °C. All strains were cultivated either in darkness or under different light, including blue, red, or white light. The surface of the culture was irradiated from the top of PDA agar plates using a panel of blue, red, or white light-emitting diodes (LEDs) at an intensity of 450 lx.

### 2.2. Plasmid Construction

The web tool CRISPOR (http://crispor.tefor.net, accessed on 15 May 2022) was used to design sgRNAs that targeted the *wc-2* gene. The pU6-*wc-2* sgRNA1 and pU6-*wc-2* sgRNA2 sequences were synthesized by Sangon Biotech Corp. and ligated into the pUC57 plasmid, generating the *wc-2* sgRNA expression plasmids pUC57-PU6-*wc-2*-sgRNA1 and pUC57-PU6-*wc-2*-sgRNA2, respectively. To construct the *wc-2* overexpression plasmid pJW-EXP-*wc-2*, the *wc-2* gene from *G. lingzhi* was first amplified using genomic polymerase chain reaction (PCR), sequenced, digested with *Sma*I and *Nhe*I, and finally ligated into the *Sma*I/*Nhe*I sites of the pJW-EXP vector [39]. The primers are listed in Table S1.

### 2.3. G. lingzhi Strain Construction

The fungal transformation of *G. lingzhi* was performed following the established method [19]. To obtain the Δ*wc-2* strain, the plasmids pUC57-PU6-*wc-2*-sgRNA1 and pUC57-PU6-*wc-2*-sgRNA2 were transformed into protoplasts of the *G. lingzhi* Cas9 strain and screened on CYM plates with 250 mg/L hygromycin B. The *wc-2* gene was subsequently cloned from the genomic DNA and sequenced to verify the gene deletion. To construct the OE*wc-2* strain, the plasmid pJW-EXP-*wc-2* was introduced into protoplasts of the wild-type *G. lingzhi* strain, which were then selected on CYM plates containing 2 mg/L carboxin. Positive transformants were then confirmed using genomic PCR. The primers are listed in Supplementary data Table S1.

### 2.4. Determination of Mycelial Growth, GA Content, Intermediate Accumulation, and Sporulation Analysis

*G. lingzhi* mycelia were scraped from the surfaces of PDA plates, washed thrice with distilled H_2_O, and dried at 45 °C until the weight remained constant. The mycelial dry weight was quantified using the gravimetric method. Squalene, lanosterol, total GAs, and individual GAs were extracted and analyzed as previously described [18,40]. A scanning electron microscope (Tescanveg3, Brno, Czech Republic) was used to observe the asexual spore morphology of the samples. To quantify asexual spore numbers, equal-sized sections of mycelium were excised from the solid medium and subsequently rinsed with PBS buffer. Equal volumes of the asexual spore suspension were prepared for analysis. The number of asexual spores was determined using a hemacytometer on an inverted optical microscope.

### 2.5. Quantitative Real Time-Polymerase Chain Reaction (qRT-PCR) Analysis

RNA was extracted using Trizol reagent (Invitrogen, Waltham, MA, USA), processed with DNase I (Fermentas, Burlington, ON, Canada), and reverse-transcribed to cDNA using the PrimeScript™ RT reagent kit (Takara, Beijing, China). qRT-PCR was conducted as previously described, with transcript levels normalized to the 18S rRNA gene [41]. The gene expression levels from engineered strains were presented as fold changes relative to the control strain, which was set as 1.0. The primers are detailed in Table S1.

### 2.6. Statistical Analysis

The results were presented as mean ± standard deviation (SD) from three biological replicates per experiment. Statistical significance was determined using the Student’s *t* test (*p* < 0.05).

## 3. Results

### 3.1. GA Accumulation in G. lingzhi Under Different Light Conditions

**Figure 1:**
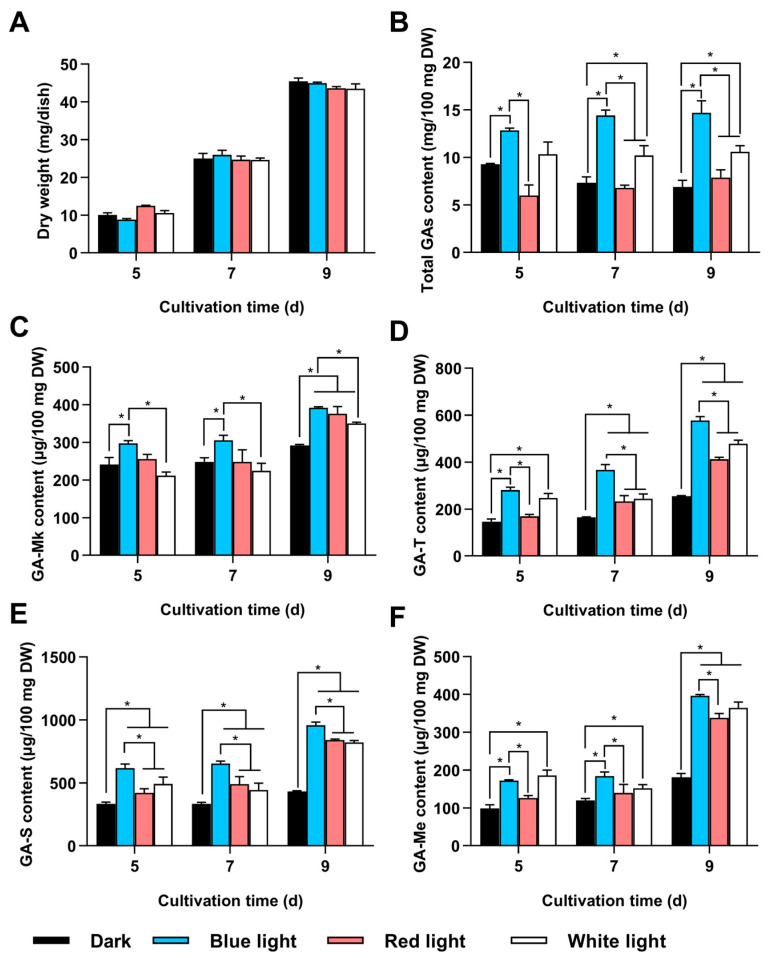
Effects of different light conditions on GA content. *G. lingzhi* CGMCC 5.616-1 was cultivated on PDA plates under four different light conditions: darkness, blue light, red light, and white light. (**A**) Dry weight; (**B**) total GA content; and levels of (**C**) GA-Mk, (**D**) GA-T, (**E**) GA-S, and (**F**) GA-Me were measured. The values represent the mean ± standard deviation of three biological replicates. Significant differences were assessed for the strain under dark and other light conditions, as well as blue light and other light conditions (* *p* < 0.05).

To investigate the effect of varying light conditions on the GA content of *G. lingzhi*, CGMCC 5.616-1 was cultivated on PDA plates under four different light conditions: darkness, blue light, red light, and white light. No significant differences in mycelia growth were observed under all the tested conditions (Figure 1A). Although the GA content was increased under all light conditions compared to darkness, the increase was most pronounced under blue light. The total GA content under blue light was 2.1 times higher than that in darkness, reaching 14.7 mg per 100 mg dry weight (DW) (Figure 1B). Moreover, the strain produced the greatest amounts of individual GAs, i.e., GA-Mk, GA-T, GA-S, and GA-Me, under blue light, yielding 392, 577.2, 957.8, and 396 μg per 100 mg DW, respectively. These amounts were 1.3, 2.3, 2.2, and 2.2 times higher than those produced in darkness (Figure 1C–F). The results demonstrate that blue light was more effective in promoting GA biosynthesis compared to other light conditions.

### 3.2. Identification and Deletion of wc-2 in G. lingzhi

**Figure 2:**
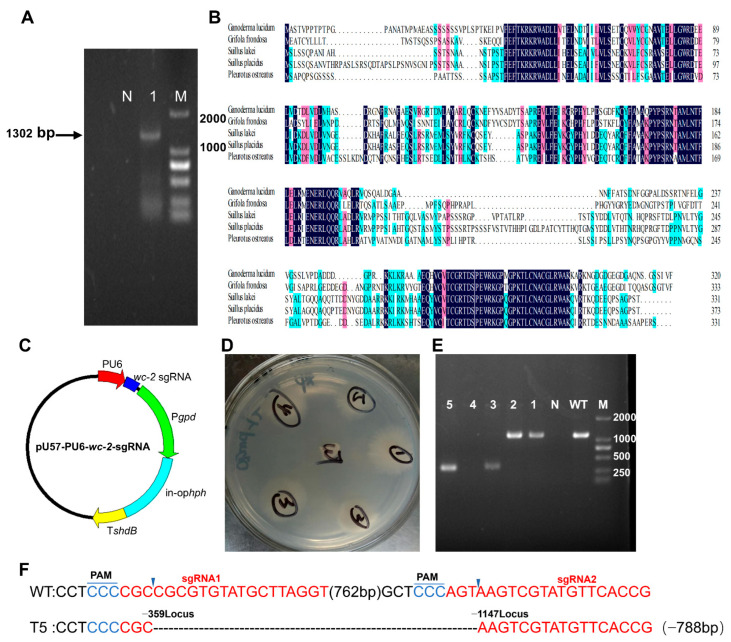
Identification and deletion of *wc-2* in *G. lingzhi*. (**A**) Amplification of the *wc-2* gene from the *G. lingzhi* genome. (**B**) Multiple alignments of amino acid sequences of *G. lingzhi* WC-2 and homologous proteins including *Grifola frondosa* (BA020283.1), *Suillus lakei* (KAG1754311.1), *Suillus placidus* (KAG183264.1), and *Pleurotus ostreatus* (XP_036636392.1). Alignments were generated using ClustalW. (**C**) pUC57-pU6-*wc-2*-sgRNA plasmid used for Δ*wc-2* construction. (**D**) Selection of Δ*wc-2* transformants on CYM plates. (**E**) Identification of positive Δ*wc-2* transformants using genomic PCR. (**F**) TA-cloning of *wc-2* deletion in selected transformant 5. sgRNA-guiding sequences are highlighted in red. WT, wild-type strain; T5, Δ*wc-2* strain.

The gene *gl24708* was identified as a homolog of WC-2 in *G. lingzhi* by querying its genome using the WC-2 amino acid sequence of *Grifola frondosa* (BA020283.1). This gene was then cloned and found to be 1302 bp in length, featuring an open reading frame of 960 bp that encoded a protein comprising 320 amino acids (Figure 2A). Protein sequence analysis indicated that *gl24708* shares a high sequence identity with WC-2 proteins from other *Basidiomycetes*, including *Grifola frondosa* (61.69%), *Suillus lakei* (46.87%), *Suillus placidus* (45.14%), and *Pleurotus ostreatus* (47.44%) (Figure 2B).

To gain insight into the function of WC-2, a *wc-2* null mutant (Δ*wc-2*) was generated in *G. lingzhi*. The constructed pUC57-pU6-*wc-2*-sgRNA1 and pUC57-pU6-*wc-2*-sgRNA2 plasmids were initially transformed into the protoplasts of the *G. lingzhi* Cas9 strain using a dual sgRNA-mediated CRISPR/Cas9 method (Figure 2C). Candidate transformants were selected from CYM plates with 250 mg/L hygromycin B and verified using genomic PCR (Figure 2D). The PCR bands amplified from transformants 3 and 5 were approximately 300 bp, which was about 800 bp shorter than the wild-type (WT) strain, corresponding to the designed sgRNA (Figure 2E). Sequencing analysis confirmed the desired deletion of the *wc-2* fragment between sgRNA1 and sgRNA2 (Figure 2F). Together these results illustrate the successful construction of the *G. lingzhi* Δ*wc-2* strain.

### 3.3. Deletion of wc-2 Significantly Reduced GA Biosynthesis

**Figure 3:**
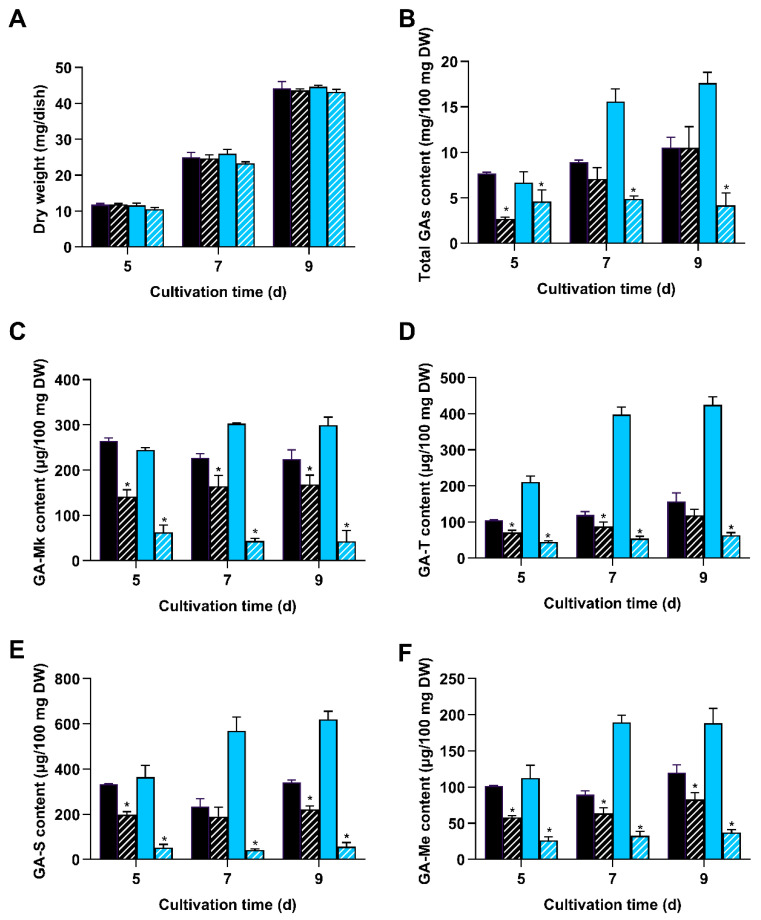
Deletion of *wc-2* significantly reduced GA content. Δ*wc-2* (hatched fill) and WT (solid fill) were cultivated on PDA plates in darkness (black) and under blue light (blue), respectively. (**A**) Dry weight; (**B**) total GA content; and levels of (**C**) GA-Mk, (**D**) GA-T, (**E**) GA-S, and (**F**) GA-Me were measured. Values represent the mean ± standard deviation of three biological replicates. Significant differences were assessed for WT and Δ*wc-2* under the same light conditions (* *p* < 0.05).

To assess if *wc-2* deletion affected GA biosynthesis in *G. lingzhi*, the Δ*wc-2* mutant and WT strain were grown on PDA plates in the darkness and under blue light, respectively. Mycelia growth and GA accumulation were then measured. No apparent differences in mycelia growth were observed under either condition, indicating that *wc-2* deletion exerted no effect on mycelia growth in *G. lingzhi* (Figure 3A). However, under blue light, the total GA content in Δ*wc-2* was 4.2 mg per 100 mg DW, representing only 23.71% of the level observed in the WT strain. In darkness, the largest decline in the total GA content of Δ*wc-2* occurred on day 5, decreasing to 34.72% of that in the WT strain (Figure 3B). Furthermore, ∆*wc-2* exhibited consistently lower individual GA contents compared to the WT strain, regardless of the light conditions. Under blue light, the maximum levels recorded for GA-Mk, GA-T, GA-S, and GA-Me were 53.54, 63.42, 56.15, and 38.65 μg per 100 mg DW, markedly decreasing to 17.54, 14.95, 9.03, and 20.21% of the levels in the WT strain, respectively. In darkness, the maximum contents of GA-Mk, GA-T, GA-S, and GA-Me were 168.06, 118.05, 221.58, and 82.55 μg per 100 mg DW, representing decreases to 67.20%, 75.64%, 65.09%, and 68.33% of the corresponding values in the WT strain, respectively (Figure 3C–F). The above results demonstrate that the deletion of *wc-2* in *G. lingzhi* leads to a significant reduction in GA content, especially under blue light.

**Figure 4:**
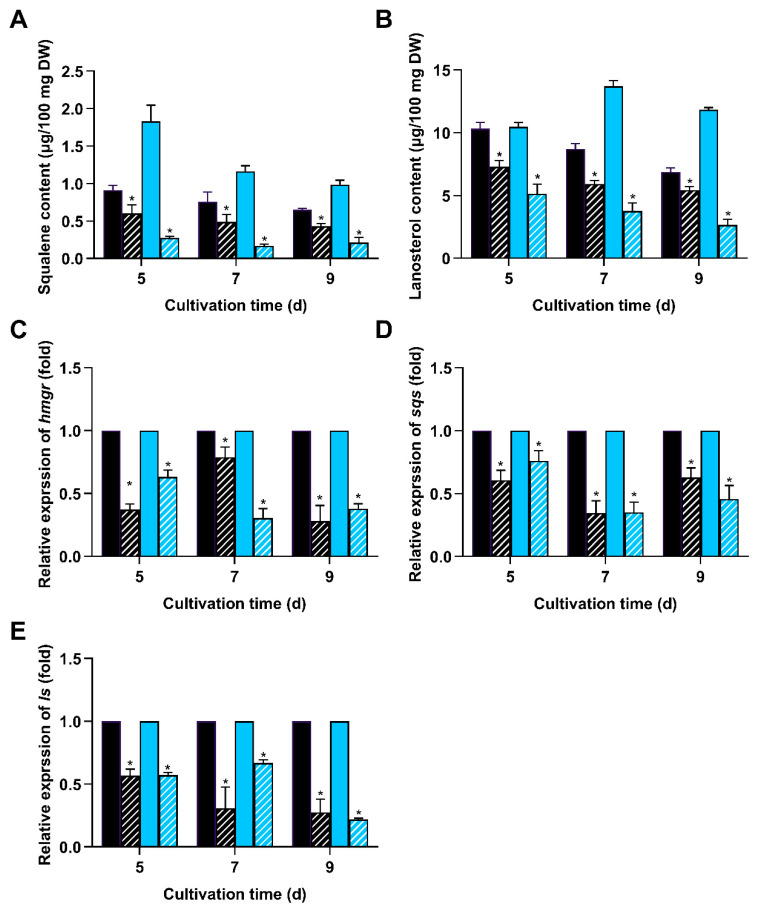
Deletion of *wc-2* reduced intermediate accumulation and gene expression in GA biosynthesis. Δ*wc-2* (hatched fill) and WT (solid fill) were cultivated on PDA agar plates in darkness (black) and blue light (blue). Contents of (**A**) squalene and (**B**) lanosterol were measured. Relative expression levels of (**C**) *hmgr*, (**D**) *sqs*, and (**E**) *ls* were analyzed using qRT-PCR. Gene expression levels in Δ*wc-2* were normalized to the corresponding levels at the same time points and light conditions in the WT strain. Values represent the mean ± standard deviation of three biological replicates. Significant differences were assessed between WT and Δ*wc-2* under the same light conditions (* *p* < 0.05).

To further understand the regulatory mechanism of WC-2 on the GA biosynthetic pathway, the contents of two important intermediate metabolites (lanosterol and squalene) and the expression of critical genes involved in GA biosynthesis were detected. ∆*wc-2* showed reduced levels of squalene and lanosterol compared to the WT strain in darkness and under light. When exposed to blue light, the contents of squalene and lanosterol were 0.276 and 5.13 μg per 100 mg DW, representing a significant decline to 15.08% and 49.04% of WT levels, respectively (Figure 4A,B). Additionally, the minimum mRNA levels of key GA biosynthetic genes (*hmgr*, *ls*, and *sqs*) in the ∆*wc-2* strain were downregulated to 27.5, 27.4, and 34.33% of those in WT in darkness and 30, 21.67, and 33.31% under blue light, respectively (Figure 4C–E). These results demonstrate that WC-2 functions as a positive regulator of the GA biosynthetic pathway.

### 3.4. Deletion of wc-2 Significantly Impaired Sporulation

**Figure 5:**
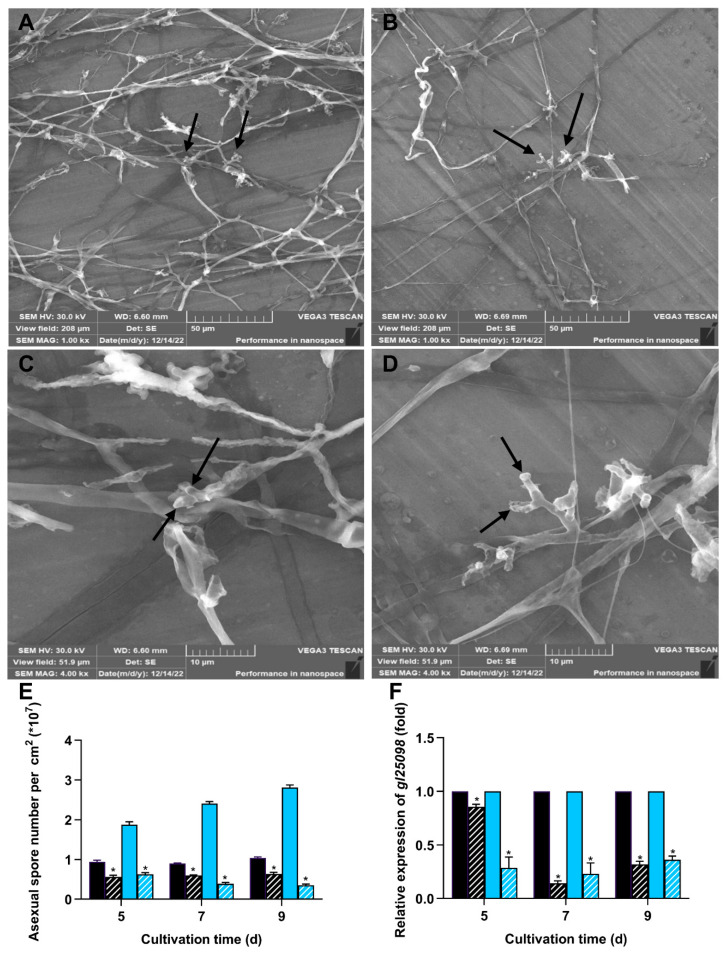
Effect of *wc-2* deletion on sporulation in *G. lingzhi*. Asexual spores in Δ*wc-2* and WT were analyzed using scanning electron microscopy. (**A**) Δ*wc-2* and (**B**) WT under 1000× magnification; (**C**) Δ*wc-2* and (**D**) WT under 4000× magnification. (**E**) Asexual spore numbers and (**F**) transcription analysis of *gl25098* in Δ*wc-2* (hatched fill) and WT (solid fill) in darkness (black) and under blue light (blue). The values represent the mean ± standard deviation of three biological replicates. Significant differences were assessed between WT and Δ*wc-2* under the same light conditions (* *p* < 0.05).

To investigate how the deletion of *wc-2* affected sporulation, we examined the production of asexual spores as well as the expression of the sporulation-specific gene *gl25098* [9]. Both WT and ∆*wc-2* strains formed aerial mycelia and asexual spores when cultured on PDA plates, which displayed identical morphological characteristics, as previously reported by Zhang and Zhong [36] (2010) (Figure 5A–D). However, in darkness and under blue light, the levels of sporulation in ∆*wc-2* were consistently lower than those of the WT strain. On day 9, the numbers of asexual spores in ∆*wc-2* were 0.64 × 10^7^ in darkness and 0.35 × 10^7^ spores/cm^2^ under blue light, respectively, corresponding to 61.53% and 13.89% of the levels observed in the WT strain (Figure 5E). Likewise, the mRNA level of *gl25098* was significantly downregulated in ∆*wc-2* both in darkness and under blue light, reaching only 31.96% and 36.1% of the levels in the WT strain, respectively (Figure 5F). These findings indicate that WC-2 also exerted an important effect on asexual sporulation in *G. lingzhi*.

### 3.5. Overexpression of wc-2 Enhanced GA Content in G. lingzhi

**Figure 6:**
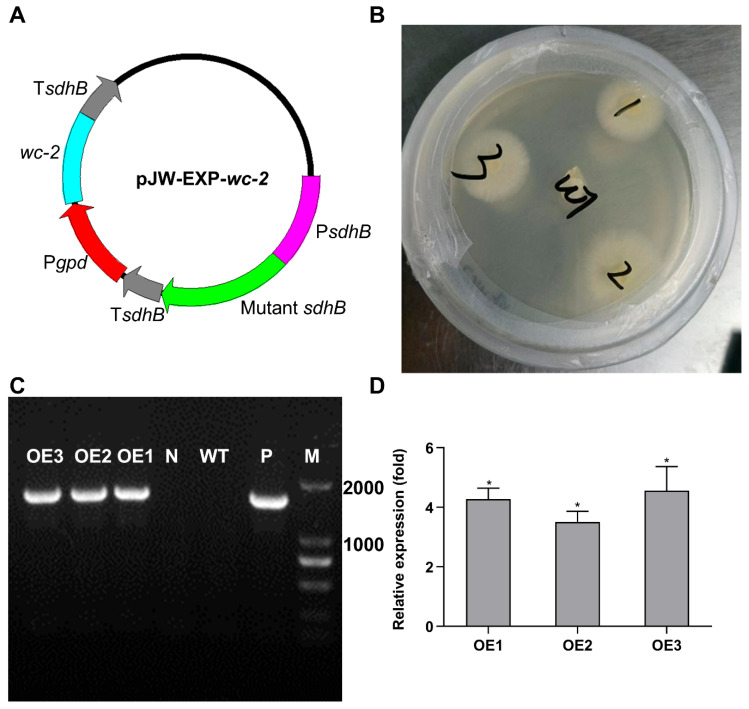
Construction of *G. lingzhi* overexpressing *wc-2*. (**A**) Schematic representation of plasmid pJW-EXP-*wc-2* used for constructing the OE*wc-2* strain. (**B**) Selection of *G. lingzhi* overexpressing *wc-2* transformants on CYM plates. (**C**) Identification of OE*wc-2* transformants using genomic PCR. M, DNA marker; P, positive control; WT, wild-type strain; N, negative control; OE1–OE3, positive transformants. (**D**) Relative transcriptional levels of *wc-2* in *G. lingzhi* overexpressing *wc-2*. Gene expression levels in *G. lingzhi* overexpressing *wc-2* were normalized to the corresponding levels in the WT strain. Values represent the mean ± standard deviation of three biological replicates. Significant differences were assessed between WT and *G. lingzhi* overexpressing *wc-2* (* *p* < 0.05).

The pJW-EXP-*wc-2* plasmid, containing a *wc-2* expression cassette driven by the constitutive glyceraldehyde-3-phosphate dehydrogenase promoter, was successfully constructed and transformed into the protoplasts of the monokaryotic CGMCC 5.616-1 strain (Figure 6A). Three positive transformants, designated OE1, OE2, and OE3, were selected on CYM plates supplemented with 2 mg/L carboxin. The transformants were subsequently identified using genomic PCR, which showed a 1700 bp band consistent with the positive control, indicating the successful insertion of the *wc-2* expression cassette into the genomic DNA (Figure 6B,C). As anticipated, the mRNA levels of *wc-2* in the three positive transformants were markedly elevated, showing increases of 4.28-, 3.5-, and 4.56-fold compared to those of the WT, respectively (Figure 6D). The total GA contents in OE1, OE2, and OE3 reached 6.93, 7.70, and 7.91 mg per 100 mg DW, which were 1.17, 1.30 and 1.33 times higher than that of the WT strain, respectively. The results confirmed the consistency of the overexpression of *wc-2.*

**Figure 7:**
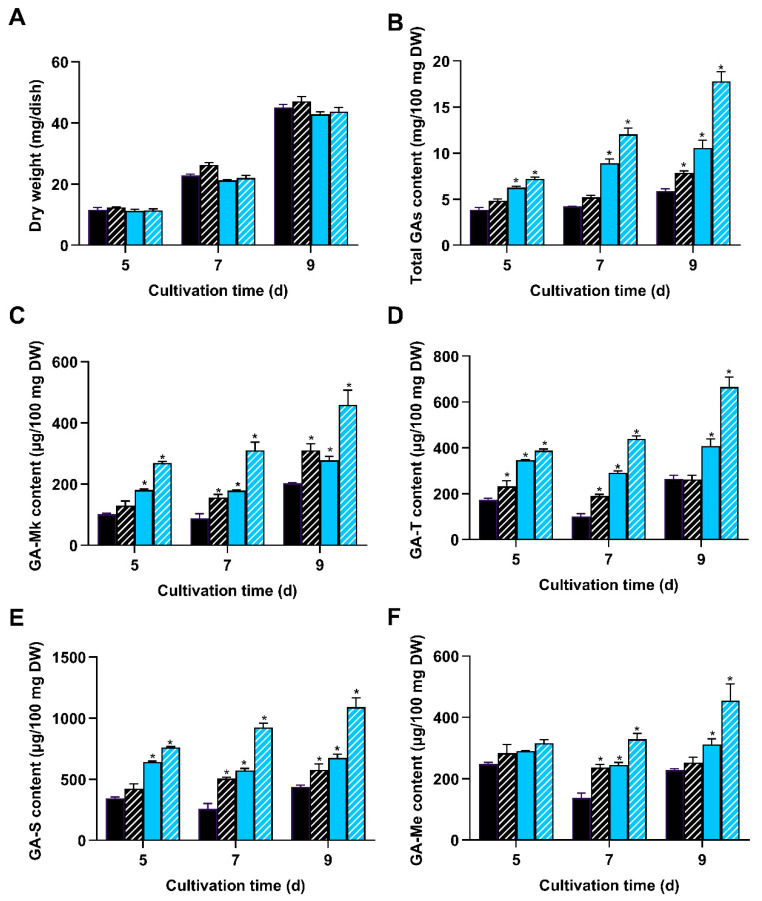
Overexpression of *wc-2* enhanced GA content in *G. lingzhi*. OEwc-2 (hatched fill) and WT (solid fill) were cultivated on PDA agar plates in darkness (black) and under blue light (blue), respectively. (**A**) Dry weight; (**B**) total GA contents; and levels of (**C**) GA-Mk, (**D**) GA-T, (**E**) GA-S, and (**F**) GA-Me were measured. The values represent the mean ± standard deviation of three biological replicates. Significant differences were assessed between WT and OE*wc-2* under the same light conditions (* *p* < 0.05).

To assess the effect of *wc-2* overexpression on GA biosynthesis, WT and OE3, hereafter referred as OE*wc-2*, were respectively cultivated on PDA plates in darkness and under blue light. Initially, there were no apparent differences in the mycelial growth among the tested light conditions, suggesting that *wc-2* overexpression had little impact on the mycelial growth (Figure 7A). Upon exposure to blue light, the total GA accumulation in OE*wc-2* reached 17.8 mg per 100 mg DW on day 9, which was 3.0 times higher than that in the WT strain grown in darkness (Figure 7B). Notably, OE*wc-2* exhibited greatly increased levels of individual GAs compared to the WT strain in darkness and under blue light. The maximum levels of GA-Mk, GA-T, GA-S, and GA-Me achieved under blue light reached 459.45, 665.19, 1090.18, and 473.53 μg per 100 mg DW, which were 2.27, 2.51, 2.49, and 2.08 times greater than those in the WT strain grown in darkness, respectively (Figure 7C–F). Altogether, these results demonstrate that the combination of *wc-2* overexpression and blue light induction markedly enhanced GA accumulation.

## 4. Discussion

In this study, the regulatory role of WC-2 in GA biosynthesis was characterized by the deletion and overexpression of *wc-2*. Deleting *wc-2* significantly impaired GA biosynthesis, while overexpressing *wc-2* resulted in increased GA accumulation. These findings highlight the critical role of the blue light receptor WC-2 in regulating the GA biosynthetic pathway in *G. lingzhi*. Numerous studies have demonstrated that WC-2 plays a crucial role in regulating secondary metabolism across various fungi, including *N. crassa*, *Cordyceps militaris*, *Sordaria fimicola*, *Mucor circinelloides*, *F. graminearum*, and *Phycomyces blakesleeanus* [34,42,43,44,45]. In addition, deletion of the *wc-2* decreased the expression of major GA biosynthetic genes and reduced the accumulation of intermediates, indicating that WC-2 functions as a positive regulator in the GA biosynthetic pathway. Previous studies have established that WC-2 is capable of regulating the expression of genes involved in secondary metabolism. In *N. crassa*, *wc-2* mutants showed impaired blue light-induced expression of carotenoid biosynthesis genes [43]. Similarly, in *Xanthophyllomyces dendrorhous*, XdWC2 regulates the expression of the phytoene desaturase gene CrtI and the astaxanthin synthase gene CrtS, both of which are crucial for astaxanthin biosynthesis [46]. Upon exposure to blue light, the WCC complex binds to the promoters of light-inducible genes [29]. It is hypothesized that WC-2 in *G. lingzhi* regulates GA biosynthesis by modulating the expression of genes involved in this metabolic pathway. Decreased transcription levels of *sqs* and *ls* may lead to lower accumulations of squalene and lanosterol in the Δ*wc-2* strain. However, the detailed mechanism requires further investigation. These results provide valuable insights into the complex regulatory network governing GA biosynthesis in response to blue light in *G. lingzhi*.

The deletion of *wc-2* led to reduced asexual spore production and the downregulated expression of the sporulation-specific gene *gl25098*, suggesting that WC-2 may participate in asexual sporulation. Previous studies have indicated that WC-2 is involved in the regulation of asexual spore production in filamentous fungi [38]. For example, a WC-2 homolog known as BLR2 was found to be essential for photoconidiation in *Trichoderma reesei* and *Trichoderma atroviride* [47,48]. Furthermore, early studies have suggested that the WCC complex may regulate transcription factors associated with spore development, thereby contributing to the formation of asexual spores [49,50,51]. However, the specific transcription factors involved in asexual sporulation in *G. lingzhi* remain unclear. Once these transcription factors are identified, a more comprehensive understanding of the mechanisms involved in spore development can be achieved.

The overexpression of *wc-2* has resulted in an increased accumulation of GAs, indicating that this approach effectively promotes GA biosynthesis. Previous research demonstrated that the overexpression of *laeA* led to 1.25- and 1.20-fold increases in GA-T and GA-Me contents, respectively, compared to the control [25]. Similarly, the addition of phenobarbital to static liquid culture yielded maximum contents of GA-Mk, GA-T, GA-S, and GA-Me of 111.9, 260.9, 123.1, and 79.4 μg per 100 mg DW, respectively. These values were 1.47, 1.28, 1.36, and 1.64 times higher than those of the control, respectively [52]. In the present study, the corresponding individual GA contents in OE*wc-2* were found to be 459.45, 665.19, 1090.18, and 473.53 μg per 100 mg DW, representing 2.27-, 2.51-, 2.49-, and 2.08-fold increases compared to the control, respectively. These values were significantly higher than the above strategies. Therefore, the strategy of overexpressing *wc-2* combined with blue light induction shows strong potential for enhancing GA biosynthesis.

## 5. Conclusions

In this study, the functional and regulatory roles of WC-2 were clarified by deleting and overexpressing *wc-2*. The blue light receptor WC-2 served as a positive regulator of GA biosynthesis and sporulation. Deleting *wc-2* showed decreased GA biosynthesis, which resulted from the downregulated expression of key GA biosynthetic genes, lower accumulation of intermediate metabolites, and impaired spore production. Conversely, overexpressing *wc-2* led to strongly enhanced GA content. These findings contribute to our understanding of the complex regulatory processes involved in GA biosynthesis.

## Data Availability

The original contributions presented in this study are included in the article/Supplementary Material. Further inquiries can be directed to the corresponding authors.

## Supplementary Materials

The following supporting information can be downloaded at: https://www.mdpi.com/article/10.3390/jof11090646/s1, Table S1: Primer sequences used in this study.
