# Supplementary material for: Blue Light Receptor WC-2 Regulates Ganoderic Acid Biosynthesis in Ganoderma lingzhi
Source: J Fungi (Basel). 2025 Sep 1;11(9):646. doi: 10.3390/jof11090646 (PMC12470545; doi:10.3390/jof11090646)
Supplement: Supplementary file 1 [file jof-11-00646-s001.zip › jof-3788990-supplementary.pdf]

Supplementary information, Table S1

Primer sequences used in this study

Primer name	Primer sequence (5'-3')	Usage
wc-2-target1-F	ACCTAAGCATACACGCGGCGGTTTTAGAGCTAGAAATAGCAAGTT	Construction of the *wc-2* sgRNA expression plasmids
wc-2-target1-R	CGCCGCGTGTATGCTTAGGTAACGGTTGAGGGGGACTTAGG	
wc-2-target2-F	AGTAAGTCGTATGTTCACCGAACGGTTGAGGGGGACTTAGG	
wc-2-target2-R	CGGTGAACATACGACTTACTGTTTTAGAGCTAGAAATAGCAAGTT	
pUC57-F	AAGCATAAAGTGTAAAGCCTGGG	
pUC57-R	CCAGGCTTTACACTTTATGCT	
pUC57-PU6-F	GCACAGATGCGTAAGGAGAAAAT	
PU6-sgRNA-R	ACTCGGTGCCACTTTTTCAAGTTG	
wc-2-cx-F	GCTCAACGACACCATCATCCT	Verification of the Δ*wc-2* strain
wc-2-cx-R	GAACCACTGTTCTGCGCTCC	
wc-2-NheI-F	GCTAGC ATGGCCTCCACCGTCCC	Construction of the pJW-EXP-*wc-2* plasmid
wc-2-SmaI-R	GGGCCCCTAGAAGACGATACTGGAACCACTG	
gpd-cx-F	TTCATCCCCCTCTCAAC	Verification of the OE*wc-2* strain
ter-cx-R	GCTCTATGTCTTGCCTTGTCTCG	
ls-qPCR-F	CTTCCGCAAGCACTACCCG	qRT-PCR
ls-qPCR-R	AGCAGATGCCCCACGAGCC	
gl25098-qPCR-F	ATACAAGGCCACCCTCCACAA	
gl25098-qPCR-R	TTGTGCTCCGCGAGGTACT	
hmgr-qPCR-F	TCCCGTGGAGCCTATTACC	
hmgr-qPCR-R	GGATGACCTCTTCGTCGTTC	
18s-qPCR-F	TATCGAGTTCTGACTGGGTTGT	
18s-qPCR-R	ATCCGTTGCTGAAAGTTGTAT	
sqs-qPCR-F	TGACGCTTCCTGACGAGA	
sqs-qPCR-R	GTGGCAGTAGAGGTTGTA	
